# Supplementary material for: The Systems Biology Research Tool: evolvable open-source software
Source: BMC Syst Biol. 2008 Jun 29;2:55. doi: 10.1186/1752-0509-2-55 (PMC2446383; doi:10.1186/1752-0509-2-55)
Supplement: Additional file 1 — SBRT Archive. An archive of the current version of the Systems Biology Research Tool. [file 1752-0509-2-55-S1.zip › sbrt-1.4.0/doc/developers_guide/api/sbrt/shell/proc/graph_theory/RedundantCycleRemoval.html]

RedundantCycleRemoval


|  |  |  |
| --- | --- | --- |
| **Overview** \| **Package** \| **Class** \| **Use** \| **Tree** \| **Deprecated** \| **Index** \| **Help** | |  |
| PREV CLASS   NEXT CLASS | **FRAMES**    **NO FRAMES**     **All Classes** |  |
| SUMMARY: NESTED \| FIELD \| CONSTR \| METHOD | DETAIL: FIELD \| CONSTR \| METHOD |  |


---


## sbrt.shell.proc.graph\_theory Class RedundantCycleRemoval

```
java.lang.Object
  sbrt.shell.proc.graph_theory.RedundantCycleRemoval
```

---

``` public class RedundantCycleRemoval extends java.lang.Object ```

This class is used

**Author:**
:   This class was written and documented by Jeremiah Wright while in the
    Wagner lab.

---

**Constructor Summary**

`RedundantCycleRemoval(java.lang.String sbmlFileName)`
  
          Constructs a new SBML file reader.


| **Method Summary** | |
| --- | --- |
| `void` | `writeFluxBounds(java.lang.String fluxBoundsFileName)`             Writes the flux bounds contained in the SBML file to a file with the provided name. |
| `void` | `writeFluxVector(java.lang.String fluxVectorFileName)`             Writes the flux vector contained in the SBML file to a file with the provided name. |
| `void` | `writeGenes(java.lang.String geneFileName)`             Writes the genes contained in the SBML file to a file with the provided name. |
| `void` | `writeRxns(java.lang.String rxnFileName)`             Writes the reactions contained in the SBML file to a file with the provided name. |

**Methods inherited from class java.lang.Object**

`clone, equals, finalize, getClass, hashCode, notify, notifyAll, toString, wait, wait, wait`

**Constructor Detail**

### RedundantCycleRemoval

```
public RedundantCycleRemoval(java.lang.String sbmlFileName)
```

:   Constructs a new SBML file reader.

    **Parameters:**: `sbmlFileName` - the name of the SBML file to be read.


**Method Detail**

### writeRxns

```
public void writeRxns(java.lang.String rxnFileName)
```

:   Writes the reactions contained in the SBML file to a file with the
    provided name.

    :   **Parameters:**: `rxnFileName` - the name of the file to generated.

---


### writeGenes

```
public void writeGenes(java.lang.String geneFileName)
```

:   Writes the genes contained in the SBML file to a file with the
    provided name.

    :   **Parameters:**: `geneFileName` - the name of the file to generated.

---


### writeFluxVector

```
public void writeFluxVector(java.lang.String fluxVectorFileName)
```

:   Writes the flux vector contained in the SBML file to a file with the
    provided name.

    :   **Parameters:**: `fluxVectorFileName` - the name of the file to generated.

---


### writeFluxBounds

```
public void writeFluxBounds(java.lang.String fluxBoundsFileName)
```

:   Writes the flux bounds contained in the SBML file to a file with the
    provided name.

    :   **Parameters:**: `fluxBoundsFileName` - the name of the file to generated.


---


|  |  |  |
| --- | --- | --- |
| **Overview** \| **Package** \| **Class** \| **Use** \| **Tree** \| **Deprecated** \| **Index** \| **Help** | |  |
| PREV CLASS   NEXT CLASS | **FRAMES**    **NO FRAMES**     **All Classes** |  |
| SUMMARY: NESTED \| FIELD \| CONSTR \| METHOD | DETAIL: FIELD \| CONSTR \| METHOD |  |


---
